# Informed by research, transformed by research

**DOI:** 10.1136/bmjebm-2023-112646

**Published:** 2023-10-19

**Authors:** Elspeth Davies

**Affiliations:** Social Anthropology, University of Cambridge, Cambridge, UK

**Keywords:** Patient Care, Medical Oncology

At university, I volunteered for a charity that raised awareness of the symptoms of cancers in young people. One afternoon, I had been asked to visit a school to talk about my experience of having been diagnosed with melanoma in situ as a teenager. My survival represented a success story, highlighting the importance of early detection. ‘Had my melanoma not been caught early, I might not be stood here to tell the tale’, I announced to the class of wide-eyed 16 year olds.

Although my treatment was deemed to be curative, the diagnosis had defined the previous few years of my life. I had been warned that I was at risk of developing more melanomas in the future. Sun cream became a permanent feature of my bag; every time the sun shone I was reminded of its carcinogenic potential. After six months of checkups, responsibility for preventing future deadly disease was placed on me. Every mark or lump became a potential threat to life, shattering the sense of trust I once felt in my body. I had further moles removed, all of which were revealed by pathology reports to be benign; my body slowly becoming a patchwork of fearful attempts to prevent a potentially deadly disease. Feeling anxious and isolated from my teenage peers, I sought solace in an online support group, where we discussed recommendations for sun hats and shared photos of new moles that concerned us.

Seven years after the initial diagnosis, I was completing my PhD as a social scientist interested in cancer early detection. Sat at the back of a windowless conference room, I heard a dermatologist present his research on melanoma overdiagnosis. He suggested most melanomas in situ were ‘overdiagnosed’—representing the unnecessary diagnosis and treatment of cells that were highly unlikely to have ever progressed to cause harm. Having been terrified by the possibility that the abnormal cells may have killed me without early detection, I became haunted by the concern that the cells might never have posed a threat to my health at all. But because my mole was removed, I will never know which is the case.

The multiplicity of my medical history is not a theoretical issue. This problem crystallises every month, when support groups post on Facebook to remind us to check our skin and I notice that a mole on my stomach has continued to change. If I am a cancer survivor, I should get it seen by my GP, who would refer me to see a specialist. If I am an overdiagnosed person, I should avoid seeking healthcare for fear that doing so is unnecessary and may lead to further redundant diagnoses. In choosing to leave a potentially concerning mole alone, I am simultaneously sparing myself superfluous medical interventions and also risking dying of metastatic disease before I turn thirty. The fracturing of my past produces a fracturing of my present and my future.

Because of my knowledge of the possibility of overdiagnosis, I am now out of place in melanoma support groups. I met up with one group in a restaurant shortly after returning from the conference where I had learned about overdiagnosis. The woman next to me talked about how she wanted to go on holiday but was worried about sun damage. She looked at me knowingly. As she reached for her drink, her wrist emerged from her sleeve to show a scar that looked familiar. It was nice to see bodies like mine—littered with scars at different stages of healing. These were risky bodies—bodies that smelt like suncream, that bore the contours of surgeons’ work as permanent features of their skin. The woman showed us a gory photo of her scar after surgery. ‘We are survivors.’ she announced. I smiled and nodded, but feared that the only thing we may have survived was overzealous medical intervention. I was now a foreigner in the community I had relied on for so long.

My peers in the support group often proudly announce that their scars ‘tell a story’. Because of my research on overdiagnosis, I no longer know what story mine tell. And yet the scars are always unavoidably present—I watch people’s eyes glance at them in meetings, at the swimming pool and even in my most intimate moments in bed. They demand an explanation that I cannot offer, representing not just the excision of malignant flesh but also the removal of the neat narrative of early detection and survival upon which I had built a life.

Because overdiagnosis only exists at the level of populations, there is no way of knowing whether my melanoma was diagnosed and treated unnecessarily or not. I must learn to live with the multiple possible stories that have been posed to me. Unlike when I was initially diagnosed, there are no resources or support groups available, and yet coming to understand myself as overdiagnosed has been as traumatic as the cancer diagnosis. When so much hope is placed on the vision of early detection, there is no space for discussing the fear that early detection may have harmed you—and the reality that you will never know if that is the case.

At the specialist conference on Preventing Overdiagnosis, there seems to be an appetite for educating people about the issue. Communicating information on the possibility of overdiagnosis before people engage with practices like cancer screening is an important part of the informed consent process, but such information will inevitably come too late for those of us who have already been diagnosed. This knowledge not only informs us, it transforms us, potentially turning heroic survivors of cancer into distrustful survivors of medical intervention. Researchers working on overdiagnosis must think carefully about the consequences of the knowledge they communicate, and consider offering support accordingly. They must go beyond *doing* research, and consider what research *does*—to people who are now patients, to their communities, and to people like me.

